# Effects of aspirin on stroke and mortality in tubercular meningitis: a meta-analysis of randomized controlled trials

**DOI:** 10.3389/fmed.2025.1682144

**Published:** 2025-11-05

**Authors:** Fang Li, Yi Zhou, Jingsi Tan, Zifei Meng, Laifa Wang, Lemei Zhu

**Affiliations:** ^1^Hunan Key Laboratory of the Research and Development of Novel Pharmaceutical Preparations, Changsha Medical University, Changsha, China; ^2^Hunan Provincial University Key Laboratory of the Fundamental and Clinical Research on Neurodegenerative Diseases, Changsha Medical University, Changsha, China; ^3^School of Public Health, Changsha Medical University, Changsha, China

**Keywords:** tubercular meningitis, aspirin, stroke, mortality, bleeding, meta-analysis

## Abstract

**Background:**

Tubercular meningitis (TBM) remains a highly lethal form of extrapulmonary tuberculosis. Aspirin, owing to its anti-inflammatory and antithrombotic properties, has been explored as adjunctive therapy, but its clinical benefits remain controversial. This meta-analysis aimed to evaluate the efficacy and safety of adjunctive aspirin in TBM, particularly its impact on stroke and all-cause mortality, and to explore the influence of different aspirin dosages.

**Methods:**

We systematically searched four databases for randomized controlled trials (RCTs) comparing adjunctive aspirin versus standard anti-tuberculosis therapy (ATT) in TBM patients. Outcomes included stroke, all-cause mortality, and bleeding events. Random-effects meta-analyses were conducted to pool risk ratios (RRs) with 95% confidence intervals (CIs). A network meta-analysis (NMA) was performed to assess the effect of different aspirin doses. The quality of evidence was assessed using the GRADE framework.

**Results:**

Five RCTs involving 580 participants were included. Adjunctive aspirin significantly reduced the risk of stroke (RR: 0.56; 95% CI: 0.33–0.95), with low-dose aspirin showing superior protective effect compared to high-dose in NMA. However, aspirin did not reduce all-cause mortality (RR: 1.00; 95% CI: 0.65–1.55) or increase bleeding risk. Sensitivity analysis indicated limited robustness of stroke outcomes, and overall evidence quality ranged from low to very low.

**Conclusion:**

Adjunctive low-dose aspirin may reduce the risk of stroke in TBM without increasing bleeding events, although it has no clear effect on mortality. Further high-quality trials are needed to confirm the optimal dosing strategy and long-term benefits of aspirin in TBM management.

**Systematic review registration:**

https://www.crd.york.ac.uk/PROSPERO/view/CRD420251110022, identifier CRD420251110022.

## Introduction

Tubercular meningitis (TBM) is a life-threatening extrapulmonary manifestation of *Mycobacterium tuberculosis* infection of the meninges (1). In 2019, approximately 164,000 adults developed TBM globally, and despite treatment, nearly 48% (about 78,200 adults) died from the disease (2). In addition to high mortality, stroke is one of the most frequent and devastating complications of TBM, occurring in 15%–30% of patients and contributing substantially to neurological disability and poor prognosis (3–6). Given this high burden of stroke, adjunctive therapies that may reduce ischemic complications are of great clinical interest. Aspirin, with its dual anti-inflammatory and antithrombotic properties, has been proposed as a potential adjunctive treatment to mitigate cerebrovascular events in TBM (7–11). However, its clinical efficacy and safety remain uncertain, warranting further systematic evaluation. In 2021, a meta-analysis by Rohilla et al. (12) included three randomized controlled trials (RCTs) comparing aspirin as an adjunctive therapy in tubercular meningitis. The results showed no significant reduction in mortality or the composite outcome (mortality and new-onset stroke) in the aspirin group compared to placebo. In recent years, additional RCTs have been published exploring the use of aspirin in TBM. Therefore, the role of aspirin in the treatment of TBM remains controversial. Considering the substantial variability in aspirin dosing across existing studies (ranging from 75 to 1000 mg), the potential dose-response relationship also warrants investigation. Thus, we aim to conduct a meta-analysis of RCTs to evaluate the effect of adjunctive aspirin in tubercular meningitis and to explore the impact of different aspirin doses. We hope that our findings will provide meaningful insight into the therapeutic management of tubercular meningitis.

## Materials and Methods

This study was conducted in accordance with the 2020 Preferred Reporting Items for Systematic Reviews and Meta-Analyses (PRISMA) guidelines (13). The study protocol was registered in PROSPERO.

### Search strategy

A comprehensive literature search was conducted on July 22, 2025, across multiple databases including PubMed, Cochrane Library, Embase, and Web of Science, using both MeSH terms and broader keywords. The detailed search strategy is provided in the Supplementary Material. Additionally, the reference lists of relevant review articles were manually screened to identify potential studies.

After removing duplicate records, two independent reviewers screened titles and abstracts to identify eligible randomized controlled trials (RCTs). The same reviewers then independently assessed the full texts of the selected articles. Eligibility criteria were defined based on the PICO framework: the population comprised patients diagnosed with tuberculous meningitis; the intervention was anti-tuberculosis treatment combined with aspirin; the comparator was anti-tuberculosis treatment alone; and the outcomes of interest included stroke, all-cause mortality, and bleeding events. Eligible patients were diagnosed with TBM based on clinical presentation (such as fever, headache, neck stiffness, altered sensorium), cerebrospinal fluid (CSF) findings (lymphocytic pleocytosis, low glucose, elevated protein), and radiological imaging (evidence of basal meningeal enhancement or infarcts), in accordance with the diagnostic definitions applied in each trial. All included patients received standard anti-tuberculosis treatment (ATT) following WHO-recommended regimens, generally consisting of isoniazid, rifampicin, pyrazinamide, and ethambutol, combined with adjunctive corticosteroids unless contraindicated.

Studies were excluded if they were not RCTs, were published in languages other than English, or if the full text was unavailable. Discrepancies in study selection were resolved through discussion with a senior author.

### Data extraction

Two independent reviewers extracted relevant data from the included studies using a standardized data extraction form. The extracted information included the first author, study design, inclusion criteria, sample size, details of the intervention and control groups, and follow-up duration. In cases of missing data, attempts were made to contact the original authors to obtain the necessary information. Any discrepancies between reviewers were resolved through discussion.

### Quality assessment

The same two reviewers independently assessed the risk of bias in the included studies using the Cochrane Collaboration’s Risk of Bias Tool (14). This tool evaluates seven domains: random sequence generation, allocation concealment, blinding of participants and personnel, blinding of outcome assessment, incomplete outcome data, selective reporting, and other potential sources of bias. Each domain was rated as having a low, unclear, or high risk of bias. Discrepancies between reviewers were resolved through discussion.

### Data extraction and outcomes

In this study, we defined low-dose aspirin as ≤150 mg/day and high-dose aspirin as ≥1000 mg/day, based on the dosing regimens used in the included RCTs. These definitions were applied consistently throughout the analyses.

The primary outcomes of this study were stroke and all-cause mortality during follow-up. Stroke was diagnosed based on clinical manifestations or confirmed by imaging.

The secondary outcome was bleeding-related events, including total bleeding events and gastrointestinal bleeding.

### Statistical analysis

All outcomes were treated as dichotomous variables, and both the number of events and total sample size were extracted. The Mantel–Haenszel (M-H) method was used to pool results, and effect estimates were presented as risk ratios (RRs) with 95% confidence intervals (CIs). Given the potential heterogeneity across studies, a random-effects model was applied to all analyses. Heterogeneity was assessed using the I^2^ statistic, interpreted as follows: 0%–40% might not be important; 30%–60% may indicate moderate heterogeneity; 50%–90% may indicate substantial heterogeneity; and 75%–100% suggests considerable heterogeneity. To evaluate the robustness of the pooled estimates, a sensitivity analysis was performed using the leave-one-out method. Given the limited number of included studies, publication bias was assessed using Egger’s test. The quality of evidence for each outcome was evaluated using the Grading of Recommendations Assessment, Development and Evaluation (GRADE) approach, with the overall certainty of evidence categorized as high, moderate, low, or very low across the five GRADE domains (15). The conventional meta-analysis was conducted using the “meta” package in R version 4.5.0. Based on its results, the pooled effects of different aspirin dosages on stroke and all-cause mortality were estimated. To further explore the optimal dosage of aspirin for tuberculous meningitis, a network meta-analysis (NMA) was performed focusing on potential beneficial outcomes. A consistency model was used if no significant inconsistency was detected (*p* > 0.05); otherwise, an inconsistency model was applied. All analyses were conducted using a random-effects model. The surface under the cumulative ranking curve (SUCRA) was used to rank the comparative effectiveness of each intervention, with SUCRA values ranging from 0 (least effective) to 1 (most effective). Sensitivity analyses were conducted to explore potential inconsistencies within the network by node-splitting method. The NMA was performed using the “netmeta” package in R version 4.5.0.

## Result

### Literature search

**FIGURE 1:**
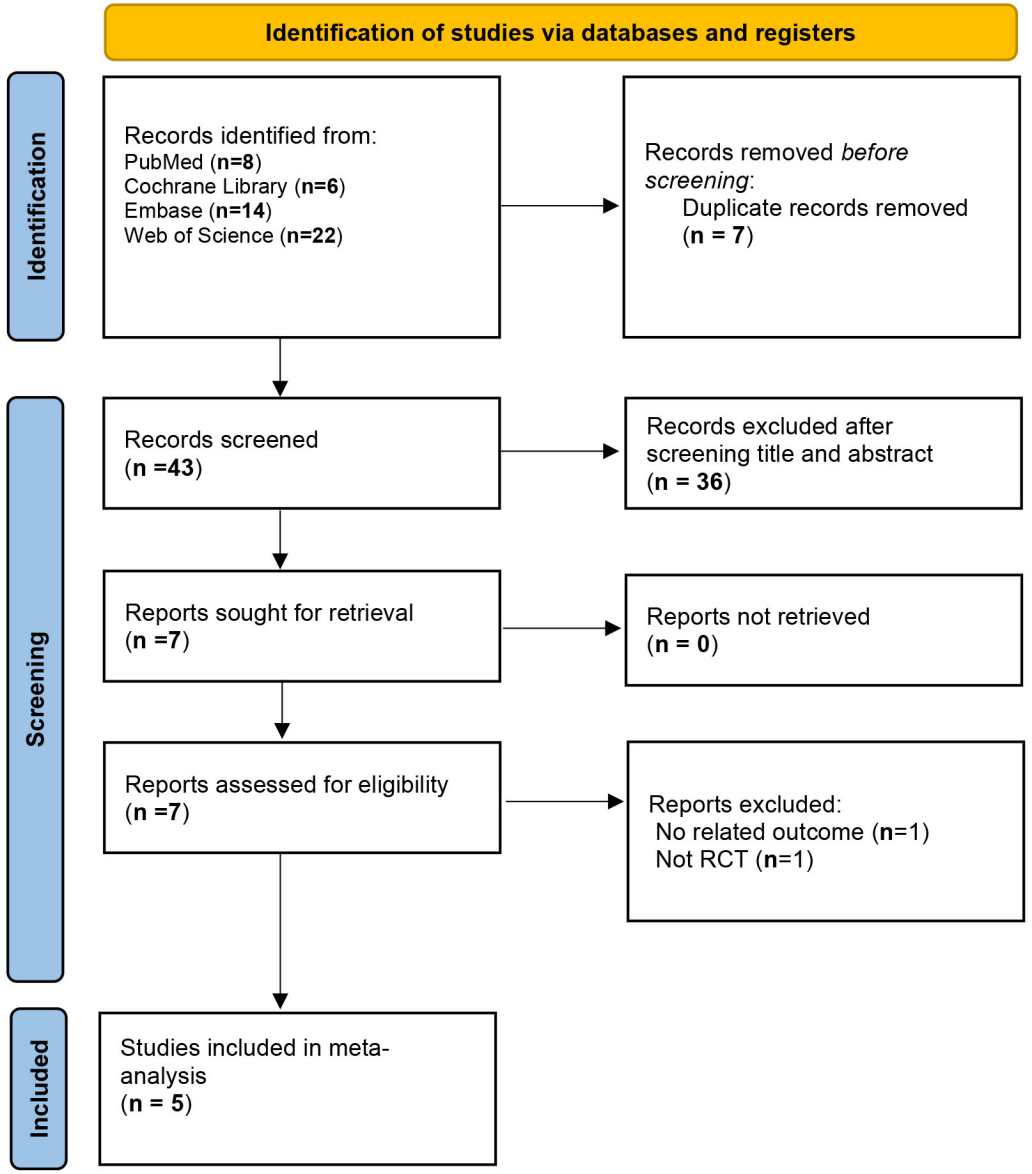
Flow chart of study selection.

A total of 50 studies were identified through the search. After removing 7 duplicate records, the titles and abstracts of the remaining 43 studies were screened. The full texts of 7 studies were then assessed for eligibility. Ultimately, 5 studies met the inclusion criteria (16–20) (Figure 1).

### Baseline study characteristic

A total of five studies comprising 580 participants were included. The aspirin dosages varied across studies. Two studies were three-arm trials comparing high-dose aspirin plus ATT, low-dose aspirin plus ATT, and ATT alone. Two studies compared low-dose aspirin plus ATT with ATT alone. One study compared high-dose aspirin plus ATT with ATT alone. Among the included studies, the trial by Schoeman et al. focused on children (Table 1).

**TABLE 1:** Study characteristic of included studies.

Study	Type of trials	Inclusion criteria	Sample size	Intervention	Control	Outcome	Follow-up duration
Bhatia et al. (16)	RCT (3 arms: aspirin, clopidogrel, placebo)	Patients (≥18 years old) with TBM diagnosed on basis of clinical, CSF, and radiological findings.	Aspirin: 77; Clopidogrel: 77; Placebo: 83.	Aspirin: standard weight-based ATT, steroids and add on treatment with aspirin 75 mg daily; Clopidogrel: standard weight-based ATT, steroids and add on treatment with clopidogrel 75 mg daily	ATT and steroids	Clinical stroke, cerebral infarction, major or minor bleeding, mortality, morbidity.	One and 3 months
Davies et al. (17)	RCT (3 arms: aspirin + additional rifampicin & linezolid, additional rifampicin & linezolid + aspirin, placebo)	Patients (≥18 years old and HIV-1 seropositivity) with a diagnosis of possible, probable, or definite TBM	Aspirin + additional rifampicin & linezolid: 16; Placebo: 20	ATT plus adjunctive 25 mg/kg rifampicin, linezolid, and aspirin (1000 mg) daily for 56 days	ATT	Stroke, death, major bleeding	56 and 180 days
Mai et al. (18)	RCT (3 arms: low dose aspirin, high dose aspirin, placebo)	Patients (≥18 years old) with suspected TBM	Low dose aspirin: 39; High dose aspirin:40; Placebo: 41.	Low dose aspirin: 81 mg aspirin and ATT; High dose aspirin: 1000 mg aspirin and ATT	ATT	Stroke, death, bleeding	60 and 240 days
Misra et al. (19)	RCT (2 arms: aspirin, placebo)	Patients with TBM diagnosed on basis of clinical, CSF, and radiological findings.	Aspirin: 59; Placebo: 59	150 mg aspirin and ATT	ATT	Death, stroke	3 months
Schoeman et al. (20)	RCT (3 arms: low dose aspirin, high dose aspirin, placebo)	Children with diagnosis of probable TBM on basis of clinical and CSF criteria	Low dose aspirin: 47; High dose aspirin: 49; Placebo: 50.	Low dose aspirin: 75 mg aspirin + ATT; High dose aspirin: 100 mg/kg aspirin + ATT	ATT	Death	6 months

### Quality assessment

**FIGURE 2:**
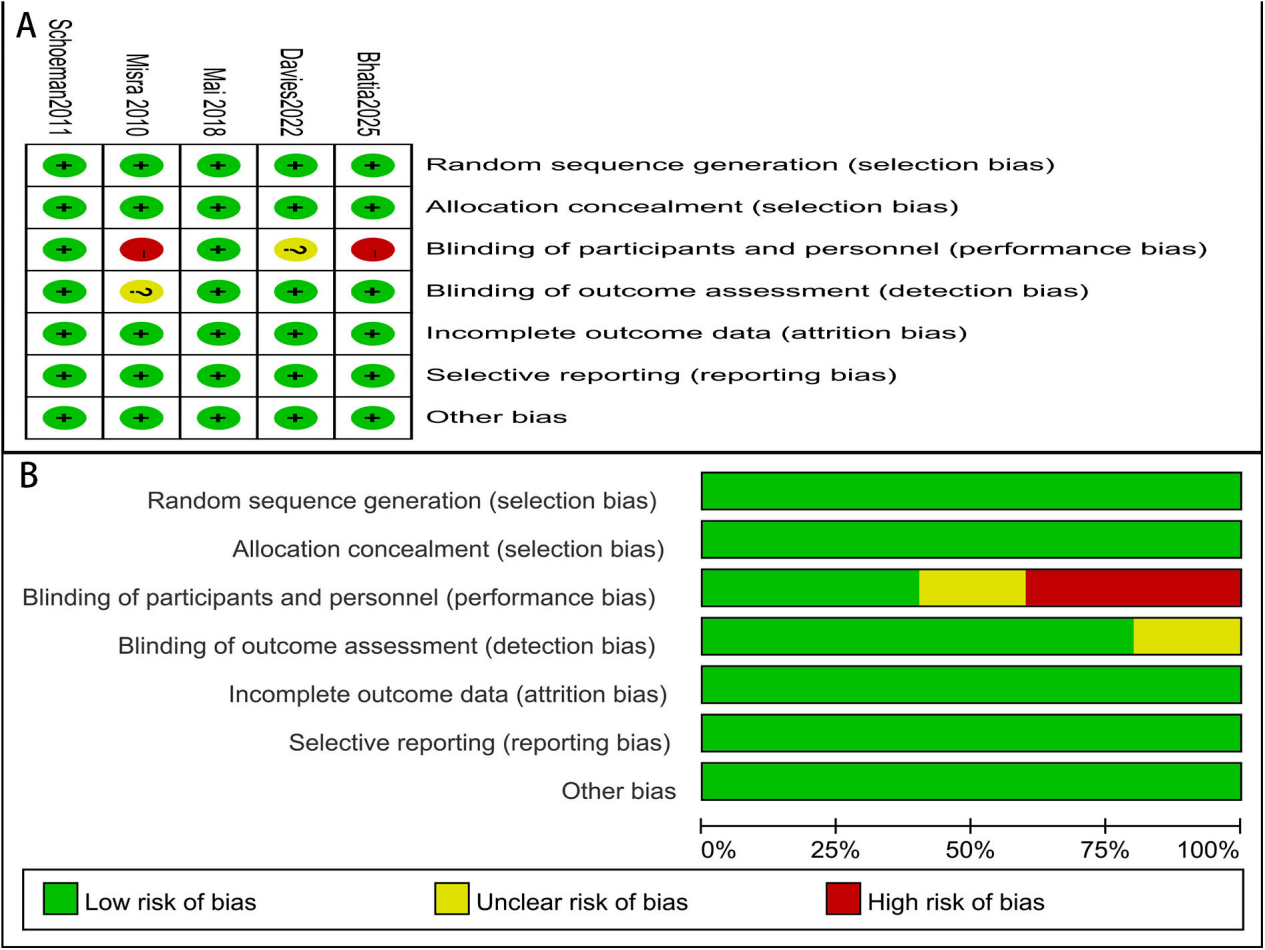
Risk of bias of each study **(A)**, and summary of risk of bias **(B)**.

Two studies were at low risk of bias. One study had an unclear risk due to insufficient information regarding blinding. Two studies were at high risk of bias because blinding was not implemented (Figure 2).

### Stroke

**FIGURE 3:**
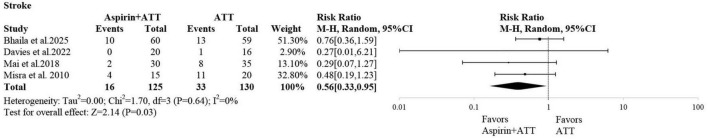
The efficacy of additional aspirin therapy on stroke.

Four studies reported stroke events. The pooled results from the conventional meta-analysis demonstrated that the addition of aspirin to anti-tuberculosis therapy (ATT) was associated with a significantly lower risk of stroke compared to ATT alone (RR: 0.56; 95% CI: 0.33–0.95) (Figure 3).

Based on these findings, adjunctive aspirin appeared to have a protective effect against stroke. However, the aspirin dosage varied widely across the included studies (ranging from 75 to 1000 mg). To further investigate the impact of different dosages, a network meta-analysis was conducted.

**FIGURE 4:**
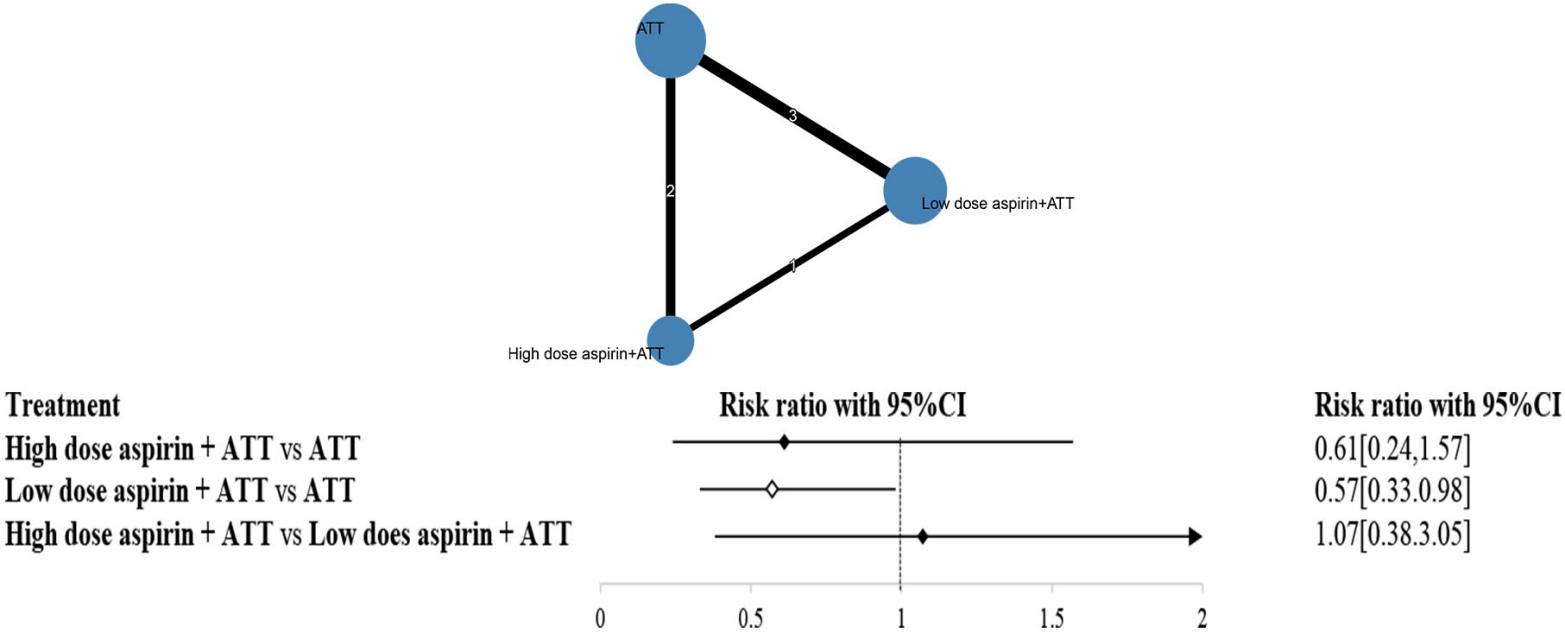
The efficacy of high dose aspirin and low dose aspirin on stroke.

Figure 4 illustrates the distribution of comparisons among three strategies: ATT alone, low-dose aspirin plus ATT, and high-dose aspirin plus ATT. The size of each circle represents the number of participants in each strategy group, while the thickness of the connecting lines and the numbers on them indicate the number of studies informing each comparison.

Inconsistency testing yielded a *p*-value of 0.6228 (>0.05), suggesting no significant inconsistency; thus, a consistency model was used for the analysis. The network meta-analysis results showed that low-dose aspirin plus ATT significantly reduced the risk of stroke compared to ATT alone (RR: 0.57; 95% CI: 0.33–0.98). However, there was no statistically significant difference between high-dose aspirin plus ATT and ATT alone (RR: 0.61; 95% CI: 0.24–1.57), nor between high-dose and low-dose aspirin groups (RR: 1.07; 95% CI: 0.38–3.05).

Surface under the cumulative ranking curve (SUCRA) values were calculated to assess the probability of each strategy being the most effective. The ranking from highest to lowest SUCRA was: low-dose aspirin plus ATT (76.56%), high-dose aspirin plus ATT (64.74%), and ATT alone (8.70%).

The node-splitting method was also employed to assess local inconsistency. All *p*-values were greater than 0.05, further confirming the absence of significant inconsistency in the network.

### Mortality

**FIGURE 5:**
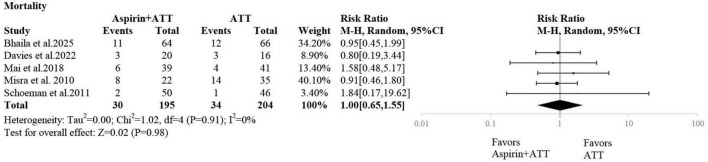
The efficacy of additional aspirin therapy on mortality.

Five studies reported mortality outcomes. The pooled analysis showed no statistically significant difference in mortality between the aspirin plus ATT group and the ATT alone group (RR: 1.00; 95% CI: 0.65–1.55) (Figure 5).

### Bleeding-related events

**FIGURE 6:**
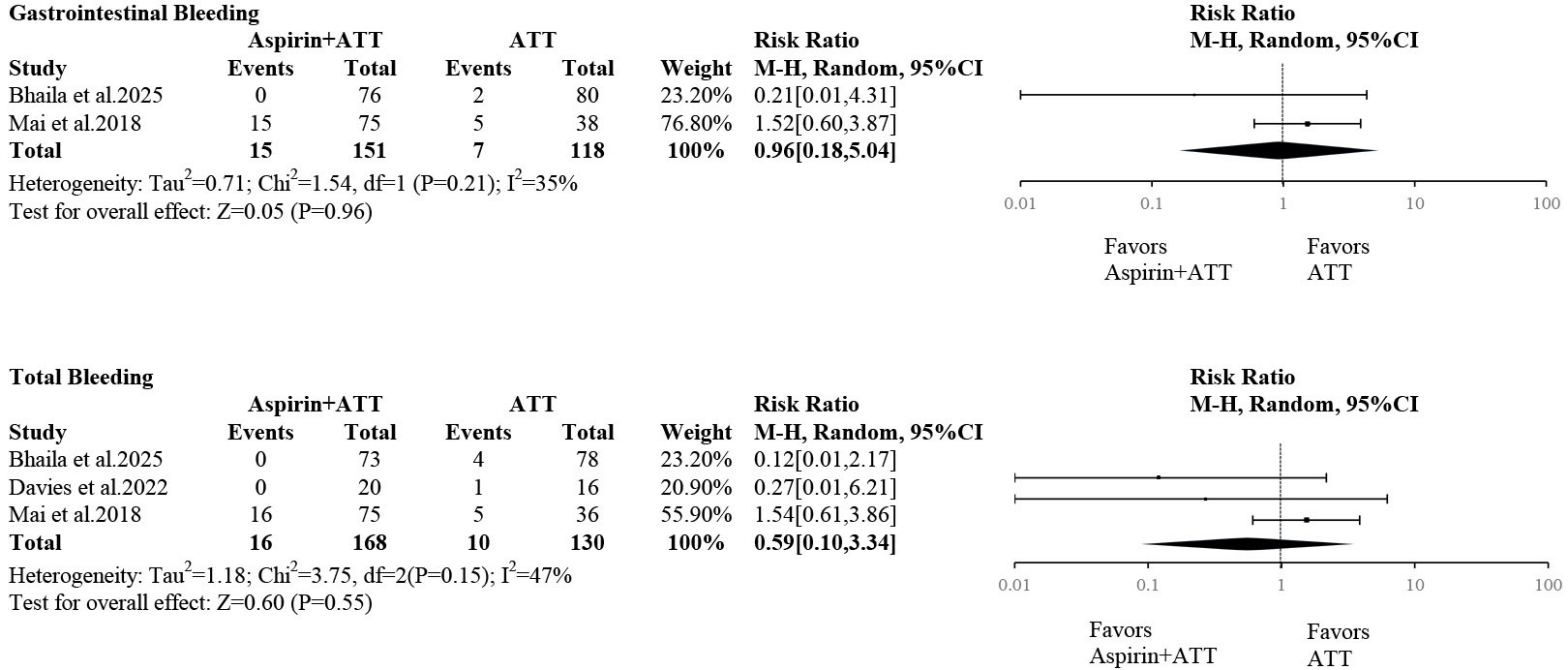
The risk of gastrointestinal bleeding and total bleeding.

Two studies reported gastrointestinal bleeding events. The pooled analysis revealed no statistically significant difference between the aspirin plus ATT group and the ATT alone group (RR = 0.96; 95% CI: 0.18–5.04) (Figure 6).

Three studies reported overall bleeding events. Similarly, the pooled results showed no statistically significant difference between the two groups (RR = 0.59; 95% CI: 0.10–3.34) (Figure 6).

### Sensitivity analysis

The leave-one-out method was employed to assess the robustness of the above outcomes. For stroke, excluding either the study by Mai et al. (18) or Misra et al. (19) resulted in the loss of statistical significance in the difference between the aspirin plus ATT group and the ATT alone group. For all other outcomes, the results remained robust (Supplementary Table 1).

### Publication bias

Except for the gastrointestinal bleeding outcome, which was based on only two studies, the Egger’s test *p*-values for all other outcomes were greater than 0.05, indicating no evidence of statistically significant publication bias (Supplementary Figure 1).

### GRADE assessment

The GRADE assessments for the above outcomes ranged from low to very low certainty (Supplementary Table 2).

## Discussion

This meta-analysis demonstrated that adjunctive aspirin significantly reduced the risk of stroke in patients with TBM, particularly when administered at low doses, without increasing bleeding risk. However, aspirin showed no effect on all-cause mortality. These findings highlight aspirin’s potential role in stroke prevention but not in improving overall survival. Our results are consistent with prior reports (12), while adding new evidence from recently published trials and incorporating a dose-specific analysis through network meta-analysis.

Regarding safety, our findings demonstrated that adjunctive aspirin did not increase the risk of gastrointestinal bleeding or total bleeding events, which is encouraging for its potential clinical use. This result supports earlier findings in similar infectious or inflammatory conditions, where low-dose aspirin did not significantly elevate bleeding risk (21). These findings suggest that aspirin can be safely administered alongside anti-tuberculosis therapy (ATT) without exacerbating bleeding risks, which are often a concern in patients with infectious diseases.

Interestingly, our analysis revealed that low-dose aspirin appeared to offer greater protective effects against stroke compared to high-dose aspirin. This is in line with previous research suggesting that lower doses of aspirin might provide sufficient anti-inflammatory and antithrombotic effects while minimizing potential adverse effects associated with higher doses (22, 23). Low-dose aspirin has also been shown to modulate eicosanoid pathways and reduce thromboxane A2 production, thereby offering cerebrovascular protection with a lower bleeding tendency (24). However, it is important to note that the results regarding stroke prophylaxis were not robust when assessed using the sensitivity analysis through the leave-one-out method. This suggests that further investigation with larger sample sizes and more consistent methodologies is required to confirm the most effective aspirin dosage for stroke prevention in TBM. Our findings suggest that high-dose aspirin did not provide additional protective effects against stroke compared with low-dose aspirin. Several mechanisms may explain this observation. First, lower doses may be sufficient to inhibit thromboxane A2–mediated platelet aggregation while minimizing adverse effects, whereas higher doses could increase bleeding tendency or interfere with prostacyclin-mediated vascular protection (25, 26). Second, pharmacodynamic studies indicate that the anti-inflammatory benefits of aspirin may plateau at lower doses, limiting the incremental efficacy of higher doses (27). Third, potential interactions between high-dose aspirin and anti-tuberculosis drugs could alter treatment response (28, 29). These factors collectively may explain why high-dose aspirin did not show superior benefits in TBM.

In 2021, Rohilla et al. conducted a meta-analysis which included only three RCTs (12). Their results showed that although this meta-analysis did not demonstrate a significant reduction in mortality or the composite outcome (mortality and new-onset stroke) with aspirin compared to placebo, there was a significant reduction in new-onset stroke in the aspirin group compared to placebo, which might be of clinical importance in the management of patients with tubercular meningitis. In contrast, our study included more studies and also found that aspirin has potential in stroke prevention, but there was no difference in mortality between the two groups. Additionally, we conducted a network meta-analysis to explore the effects of different aspirin doses on stroke prevention, and the results indicated that low-dose aspirin had a better preventive effect.

Stroke prevention remains a crucial therapeutic goal in TBM, as ischemic complications are strongly associated with long-term neurological disability and mortality (30). Given that up to one-third of TBM patients may develop stroke, adjunctive strategies to mitigate this risk are urgently needed (31). Aspirin shows promise in this regard; however, variability in patient response has been observed. One possible explanation is aspirin resistance, a phenomenon characterized by reduced platelet responsiveness to aspirin’s antiplatelet effects (32). Aspirin resistance has been reported in 10%–30% of patients in cardiovascular and neurological settings, and may also influence outcomes in TBM (32). Future studies incorporating platelet function testing or alternative antiplatelet agents could provide valuable insights into optimizing cerebrovascular protection in TBM.

To our knowledge, this is the most up-to-date meta-analysis of aspirin in TBM, including five RCTs with 580 participants. Uniquely, we applied a network meta-analysis to directly compare low- versus high-dose aspirin, providing novel evidence that low-dose aspirin may offer greater cerebrovascular protection. This dose-specific evaluation adds new insights that were not available in previous systematic reviews. However, there are several limitations to our study that should be considered. The studies included in the meta-analysis had significant variability in their design and sample sizes, which may have introduced bias or reduced the precision of our findings. Furthermore, the dosing regimens of aspirin varied widely, ranging from 75 to 1000 mg, which introduces a level of heterogeneity that may have influenced the overall results. The lack of consistency in aspirin dosing also limits the ability to draw definitive conclusions about the optimal dose for stroke prevention in TBM patients. Moreover, the studies included in this meta-analysis varied in their follow-up durations, with some reporting short-term outcomes and others providing long-term data. This inconsistency in follow-up timeframes may have contributed to the lack of significant findings regarding all-cause mortality, as longer-term outcomes might differ from those observed in the short term. There are several limitations to our study that should be considered. First, the studies included in the meta-analysis had significant variability in their design, including differences in population characteristics, aspirin dosing regimens, and follow-up durations. This variability may have introduced bias or reduced the precision of our findings, limiting the generalizability of the results. The small number of included studies (five) also constrained the power of the analysis, potentially leading to Type II errors (failing to detect a true effect) in some of the secondary outcomes, such as mortality and bleeding events. Second, the dosing regimens of aspirin varied widely across the included studies, ranging from 75 to 1000 mg. This inconsistency in aspirin dosage creates heterogeneity that makes it difficult to establish a definitive optimal dose for stroke prevention in TBM patients. The lack of a standard protocol for aspirin dosing also raises concerns about the comparability of the studies, making it challenging to draw firm conclusions on the best therapeutic approach. Third, while we used rigorous statistical methods, including network meta-analysis and sensitivity analysis, the results related to stroke prophylaxis were not entirely robust when assessed through the leave-one-out method. This indicates that the observed benefits of aspirin on stroke risk could be influenced by specific studies in the meta-analysis. Larger studies or multi-center trials are needed to validate these findings and provide a more reliable estimate of the treatment effect. Furthermore, the studies included in this meta-analysis varied in their follow-up durations. Some studies assessed only short-term outcomes, while others provided long-term data, introducing a potential source of bias. This difference in follow-up timeframes may have contributed to the lack of significant findings regarding all-cause mortality, as the effects of aspirin might only become evident over longer periods. Finally, the quality of evidence for some of the outcomes, as assessed by the GRADE approach, ranged from low to very low. This suggests that the findings should be interpreted with caution and highlights the need for further research with improved study designs and larger sample sizes.

## Conclusion

While our study supports the use of low-dose aspirin as an adjunctive therapy for stroke prevention in TBM, the lack of effect on mortality and the uncertainty regarding optimal dosing highlight the need for further randomized controlled trials to clarify these issues.

## Data Availability

The datasets presented in this study can be found in online repositories. The names of the repository/repositories and accession number(s) can be found in the article/Supplementary material.
