# Supplementary material for: Effects of aspirin on stroke and mortality in tubercular meningitis: a meta-analysis of randomized controlled trials
Source: Front Med (Lausanne). 2025 Nov 5;12:1682144. doi: 10.3389/fmed.2025.1682144 (PMC12626909; doi:10.3389/fmed.2025.1682144)
Supplement: Supplementary file 1 [file Table_1.docx]

**Supplement**

Search strategy

eTable1. Sensitivity analysis by leave-one-out method

eFigure1. Egger’s test for stroke, mortality, and total bleeding

Search strategy

PubMed (8)

1. "Tuberculosis"[Mesh] OR “Tuberculosis”[tiab] OR “Tuberculoses”[tiab]
2. "Meningitis"[Mesh] OR “"Meningitis"[tiab]” OR “Meningitides [tiab]” OR “Pachymeningitis[tiab]” OR “Pachymeningitides[tiab]”
3. "Aspirin"[Mesh] OR (Acetylsalicylic Acid[tiab]) OR (Acid, Acetylsalicylic[tiab]) OR (2-(Acetyloxy)benzoic Acid[tiab]) OR (Micristin[tiab]) OR (Solprin[tiab]) OR (Solupsan[tiab]) OR (Zorprin[tiab]) OR (Acetysal[tiab]) OR (Acylpyrin[tiab]) OR (Aloxiprimum[tiab]) OR (Colfarit[tiab]) OR (Dispril[tiab]) OR (Easprin[tiab]) OR (Ecotrin[tiab]) OR (Endosprin[tiab]) OR (Magnecyl[tiab]) OR (Polopirin[tiab]) OR (Polopiryna[tiab])
4. Rando*[tiab]
5. #1 AND #2 AND #3 AND #4

Cochrane Library (6)

1. MeSH descriptor: [Tuberculosis] explode all trees
2. (Tuberculosis OR Tuberculoses):ti,ab
3. #1 OR #2
4. MeSH descriptor: [Meningitis] explode all trees
5. (Meningitis OR Meningitides OR Pachymeningitis OR Pachymeningitides): ti,ab
6. #4 OR #5
7. MeSH descriptor: [Aspirin] explode all trees
8. (Acetylsalicylic Acid OR Acid, Acetylsalicylic OR Micristin OR Solprin OR Solupsan OR Zorprin OR Acetysal OR Acylpyrin OR Aloxiprimum OR Colfarit OR Dispril OR Easprin OR Ecotrin OR Endosprin OR Magnecyl OR Polopirin OR Polopiryna):ti,ab
9. #7 OR #8
10. (rando*): ti,ab
11. #3 AND #6 AND #9 AND #10

Embase (14)

1. 'tuberculosis'/exp
2. ((tuberculosis) OR (tuberculoses)):ti,ab
3. #1 OR #2
4. ' Meningitis '/exp
5. ((Meningitis) OR (Meningitides) OR (Pachymeningitis) OR (Pachymeningitides)):ti,ab
6. #4 OR #5
7. #3 AND #6
8. 'acetylsalicylic acid'/exp
9. ((Aspirin) OR (Acetylsalicylic Acid) OR (Acid, Acetylsalicylic) OR (2-Acetyloxy benzoic Acid) OR (Micristin) OR (Solprin) OR (Solupsan) OR (Zorprin) OR (Acetysal) OR (Acylpyrin) OR (Aloxiprimum) OR (Colfarit) OR (Dispril) OR (Easprin) OR (Ecotrin) OR (Endosprin) OR (Magnecyl) OR (Polopirin) OR (Polopiryna)):ti,ab
10. #8 OR #9
11. (rando*):ti,ab
12. #3 AND #6 AND #7 AND #10 AND #11

Web of science (22)

1. TS=(Tuberculosis OR Tuberculoses)
2. TS=( Meningitis OR Meningitides OR Pachymeningitis OR Pachymeningitides)
3. TS=(Aspirin OR Acetylsalicylic Acid OR Acid, Acetylsalicylic OR 2-(Acetyloxy)benzoic Acid OR Micristin OR Solprin OR Solupsan OR Zorprin OR Acetysal OR Acylpyrin OR Aloxiprimum OR Colfarit OR Dispril OR Easprin OR Ecotrin OR Endosprin OR Magnecyl OR Polopirin OR Polopiryna )
4. TS=(rando*)
5. #1 AND #2 AND #3 AND #4

eTable1. Sensitivity analysis by leave-one-out method

| **Stroke** | |
| --- | --- |
| **Study** | **RR [95%CI]** |
| Bhatia 2025 | 0.41 [0.19,0.88] |
| Davies 2022 | 0.57 [0.33,0.98] |
| Mai 2018 | 0.62 [0.35.1.09] |
| Misra 2010 | 0.60 [0.31,1.15] |
| **Mortality** | |
| **Study** | **RR [95%CI]** |
| Bhatia 2025 | 1.04 [0.61,1.77] |
| Davies 2022 | 1.03 [0.65,1.62] |
| Mai 2018 | 0.94 [0.59,1.49] |
| Misra 2010 | 1.07 [0.61,1.88] |
| Schoeman 2011 | 0.98 [0.63,1.53] |
| **Gastrointestinal bleeding** |  |
| **Study** | **RR [95%CI]** |
| Bhatia 2025 | 1.52 [0.60,3.87] |
| Mai 2018 | 0.21 [0.01,4.31] |
| **Total bleeding** |  |
| **Study** | **RR [95%CI]** |
| Bhatia 2025 | 1.26 [0.42,3.75] |
| Davies 2022 | 0.61 [0.05,6.21] |
| Mai 2018 | 1.54 [0.61,3.86] |

eFigure1. Egger’s test for stroke, mortality, and total bleeding


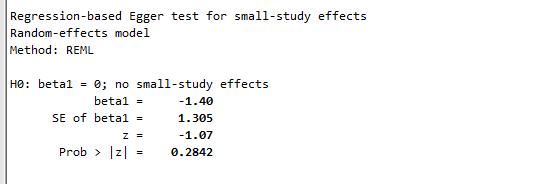


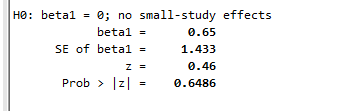


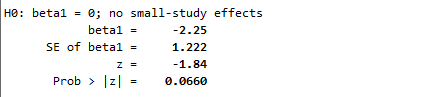


eTable2. Grade assessment

| **Quality assessment** | | | | | | | **No of patients** | | **Effect** | **Quality** |
| --- | --- | --- | --- | --- | --- | --- | --- | --- | --- | --- |
|  |  |  |  |  |  |  |  |  |  |  |
| **No of studies** | **Design** | **Risk of bias** | **Inconsistency** | **Indirectness** | **Imprecision** | **Other considerations** | **Experimental** | **Control** | **RR [95%CI]** |  |
| **Stroke** | | | | | | | | | | |
| 4 | randomised trials | serious | serious | no serious indirectness | no serious imprecision | none | 125 | **130** | **0.56[0.33,0.95]** | LOW |
| **Mortality** | | | | | | | | | | |
| 5 | randomised trials | serious | serious | no serious indirectness | no serious imprecision | none | 195 | 204 | **1.00[0.65,1.55]** | LOW |
| **Gastrointestinal bleeding** | | | | | | | | | | |
| 2 | randomised trials | serious | serious | no serious indirectness | serious imprecision | none | 151 | **118** | **0.96[0.18,5.04]** | Very  LOW |
| **Total bleeding** | | | | | | | | | | |
| 3 | randomised trials | serious | serious | no serious indirectness | serious imprecision | none | 168 | 130 | **0.59[0.10,3.34]** | Very  LOW |
