# Supplementary material for: Effects of aspirin on stroke and mortality in tubercular meningitis: a meta-analysis of randomized controlled trials
Source: Front Med (Lausanne). 2025 Nov 5;12:1682144. doi: 10.3389/fmed.2025.1682144 (PMC12626909; doi:10.3389/fmed.2025.1682144)
Supplement: Supplementary file 2 [file Table_2.docx]

Authors report a meta-analysis based on 5 studies and 580 patients on the role of aspirin on mortality. They conclude that adjunctive low-dose aspirin may reduce the risk of stroke in TBM without increasing bleeding events, although it has no clear effect on mortality. The subject is of interest and I have the following comments:
1. First paragraph of introduction is not relevant. The authors may like to present that how common is stroke in TBM and justify the role of aspirin in TBM citing the available literature

Response: Thank you for this important suggestion. We have revised the first paragraph of the Introduction to highlight the frequency of stroke in TBM and its clinical importance. We have also added references to justify the rationale for aspirin use in this context.

Action:” Tubercular meningitis (TBM) is a life-threatening extrapulmonary manifestation of Mycobacterium tuberculosis infection of the meninges. In 2019, approximately 164,000 adults developed TBM globally, and despite treatment, nearly 48% (about 78,200 adults) died from the disease. In addition to high mortality, stroke is one of the most frequent and devastating complications of TBM, occurring in 15%–30% of patients and contributing substantially to neurological disability and poor prognosis. Given this high burden of stroke, adjunctive therapies that may reduce ischemic complications are of great clinical interest. Aspirin, with its dual anti-inflammatory and antithrombotic properties, has been proposed as a potential adjunctive treatment to mitigate cerebrovascular events in TBM. However, its clinical efficacy and safety remain uncertain, warranting further systematic evaluation.” (Page3, Line49-59)

2. Method section is properly presented

Response: Thank you very much for your review and recognition.

3. Results are clearly stated

Response: Thank you very much for your review and recognition.

4. Low dose and high dose aspirin should be clearly stated

Response: We agree with the reviewer. We have clarified the definitions of “low-dose” and “high-dose” aspirin in both the Methods and Results sections to ensure consistency and clarity.

Action: “In this study, we defined low-dose aspirin as ≤150 mg/day and high-dose aspirin as ≥1000 mg/day, based on the dosing regimens used in the included RCTs. These definitions were applied consistently throughout the analyses.” (Page4, Line116-118)

5. Why high dose aspirin is not effective may be discussed or commented

Response: We appreciate this insightful suggestion. We have expanded the Discussion to explain potential reasons for the lack of efficacy of high-dose aspirin, including the possibility of increased adverse effects, altered pharmacodynamics, and the balance between antithrombotic and bleeding risks at higher doses.

Action: Our findings suggest that high-dose aspirin did not provide additional protective effects against stroke compared with low-dose aspirin. Several mechanisms may explain this observation. First, lower doses may be sufficient to inhibit thromboxane A₂–mediated platelet aggregation while minimizing adverse effects, whereas higher doses could increase bleeding tendency or interfere with prostacyclin-mediated vascular protection. Second, pharmacodynamic studies indicate that the anti-inflammatory benefits of aspirin may plateau at lower doses, limiting the incremental efficacy of higher doses. Third, potential interactions between high-dose aspirin and anti-tuberculosis drugs could alter treatment response. These factors collectively may explain why high-dose aspirin did not show superior benefits in TBM.

6. The discussion should be modified, the authors may consider stating their results in a sentence or two and discuss in then light of the published information

Response: Thank you for this helpful recommendation. We have restructured the Discussion section to briefly state our main results at the beginning, followed by a discussion in the context of existing literature.

Action: “This meta-analysis demonstrated that adjunctive aspirin significantly reduced the risk of stroke in patients with TBM, particularly when administered at low doses, without increasing bleeding risk. However, aspirin showed no effect on all-cause mortality. These findings highlight aspirin’s potential role in stroke prevention but not in improving overall survival. Our results are consistent with prior reports(12), while adding new evidence from recently published trials and incorporating a dose-specific analysis through network meta-analysis.” (Page7, Line21-227)

7. Line 23 “—the results were not fully robust” may be modified to the results were not robust

Response: We agree and have made the suggested modification.

Action: In the *Discussion* (Page 24, Line 454), we replaced “the results were not fully robust” with “the results were not robust.”

8. What is new in this study should be clearly stated

Response: Thank you for this valuable suggestion. We have added a clear statement emphasizing the novelty of our study, particularly the inclusion of the most recent RCTs and the use of network meta-analysis to evaluate aspirin dosing.

Action: “To our knowledge, this is the most up-to-date meta-analysis of aspirin in TBM, including five RCTs with 580 participants. Uniquely, we applied a network meta-analysis to directly compare low- versus high-dose aspirin, providing novel evidence that low-dose aspirin may offer greater cerebrovascular protection. This dose-specific evaluation adds new insights that were not available in previous systematic reviews.” (Page8-9, Line277-281)

9. The last sentence of conclusion may be deleted as it is not relevant to the present study
Response: We agree with the reviewer’s observation and have deleted the last sentence of the Conclusion.

Action: “While our study supports the use of low-dose aspirin as an adjunctive therapy for stroke prevention in TBM, the lack of effect on mortality and the uncertainty regarding optimal dosing highlight the need for further randomized controlled trials to clarify these issues.” (Page10, Line322-324)

This is a well written paper and after the minor changes may be favourably considered for publication.

Response: Thank you once again for your suggestion!
